# Supplementary material for: Resolving the full spectrum of human genome variation using Linked-Reads
Source: Genome Res. 2019 Apr;29(4):635–45. doi: 10.1101/gr.234443.118 (PMC6442396; doi:10.1101/gr.234443.118)
Supplement: Supplemental Material [file supp_gr.234443.118_Supplemental_longranger-2.2.zip › longranger-2.2.tar.bz2!/lr2.2-public/lib/stan/cmdstan/stan/doc/api/html/common__type_8hpp_source.html]

Stan: src/stan/math/matrix/common\_type.hpp Source File


|  |  |
| --- | --- |
|  | Stan  2.5.0  probability, sampling & optimization |


- Main Page
- Related Pages
- Namespaces
- Classes
- Files

- File List
- File Members

All Classes Namespaces Files Functions Variables Typedefs Enumerations Enumerator Friends Macros Pages

- src
- stan
- math
- matrix

common\_type.hpp

Go to the documentation of this file.

1 #ifndef STAN\_\_MATH\_\_MATRIX\_\_COMMON\_TYPE\_HPP

2 #define STAN\_\_MATH\_\_MATRIX\_\_COMMON\_TYPE\_HPP

3

4 #include <vector>

5 #include <stan/math/matrix/Eigen.hpp>

6 #include <boost/math/tools/promotion.hpp>

7

8 namespace stan {

9

10  namespace math {

11

12  template <typename T1, typename T2>

13  struct common\_type {

14  typedef typename boost::math::tools::promote\_args<T1,T2>::type type;

15  };

16

17  template <typename T1, typename T2>

18  struct common\_type<std::vector<T1>, std::vector<T2> > {

19  typedef std::vector<typename common\_type<T1,T2>::type> type;

20  };

21

22  template <typename T1, typename T2, int R, int C>

23  struct common\_type<Eigen::Matrix<T1,R,C>, Eigen::Matrix<T2,R,C> > {

24  typedef Eigen::Matrix<typename common\_type<T1,T2>::type,R,C> type;

25  };

26

27  }

28 }

29

30

31 #endif

stan::math::common\_type

**Definition:** common\_type.hpp:13

stan::math::common\_type< std::vector< T1 >, std::vector< T2 > >::type

std::vector< typename common\_type< T1, T2 >::type > type

**Definition:** common\_type.hpp:19

stan::math::common\_type< Eigen::Matrix< T1, R, C >, Eigen::Matrix< T2, R, C > >::type

Eigen::Matrix< typename common\_type< T1, T2 >::type, R, C > type

**Definition:** common\_type.hpp:24

stan::math::common\_type::type

boost::math::tools::promote\_args< T1, T2 >::type type

**Definition:** common\_type.hpp:14

Eigen.hpp


---

[ Stan Home Page ]

*© 2011–2014,
Stan Development Team.*
